# Delayed malignant melanoma recurrence simulating primary ovarian cancer: Case report

**DOI:** 10.1186/1477-7819-6-124

**Published:** 2008-11-20

**Authors:** Anastasios Boutis, Rosalia Valeri, Ippokratis Korantzis, Dimitrios Valoukas, Ioannis Andronikidis, Charalambos Andreadis

**Affiliations:** 1 3rd Department of Clinical Oncology, Theagenion Cancer Hospital, Thessaloniki, Greece; 2 Department of Oncology-Chemotherapy, 2nd "IKA" General Hospital, Thessaloniki, Greece; 3 Department of Cytopathology, Theagenion Cancer Hospital, Thessaloniki, Greece; 4 1st Department of Clinical Oncology, Theagenion Cancer Hospital, Thessaloniki, Greece; 5 Department of Radiotherapy, AHEPA University General Hospital, Thessaloniki, Greece

## Abstract

**Background:**

Metastatic involvement of the ovary from malignant melanoma is uncommon and presents a diagnostic challenge. Most cases are associated with disseminated disease and carry a dismal prognosis. Delayed ovarian recurrences from melanoma may mimic primary ovarian cancer and lead to aggressive cytoreductive procedures.

**Case presentation:**

A case of malignant melanoma in a premenopausal patient is presented with late abdominal and ovarian metastatic spread, where ascitic fluid cytology led to an accurate preoperative diagnosis and the avoidance of unnecessary surgical procedures.

**Conclusion:**

Secondary ovarian involvement is associated with a poor prognosis and efforts should be made for adequate palliation. Pathologic diagnosis with non-invasive procedures is crucial in order to avoid unnecessary surgery. Surgical interventions may be undertaken only in selected cases of limited metastatic disease, where complete resection is expected

## Background

The ovary is a frequent site of secondary spread from extra-ovarian malignancies. Approximately 6–7% of the patients presenting with suspected ovarian neoplasm will prove to suffer from metastatic disease to the ovary [1]. Besides gynecologic cancers, which tend to involve the ovaries by direct invasion, gastrointestinal adenocarcinomas, followed by breast cancer are the most common nongynecologic malignancies, which metastasize to the ovaries [1,2]. Ovarian involvement by metastatic malignant melanoma is relatively uncommon and it is rare for melanoma to present clinically as an ovarian mass [3].

## Case presentation

**Figure 1:**
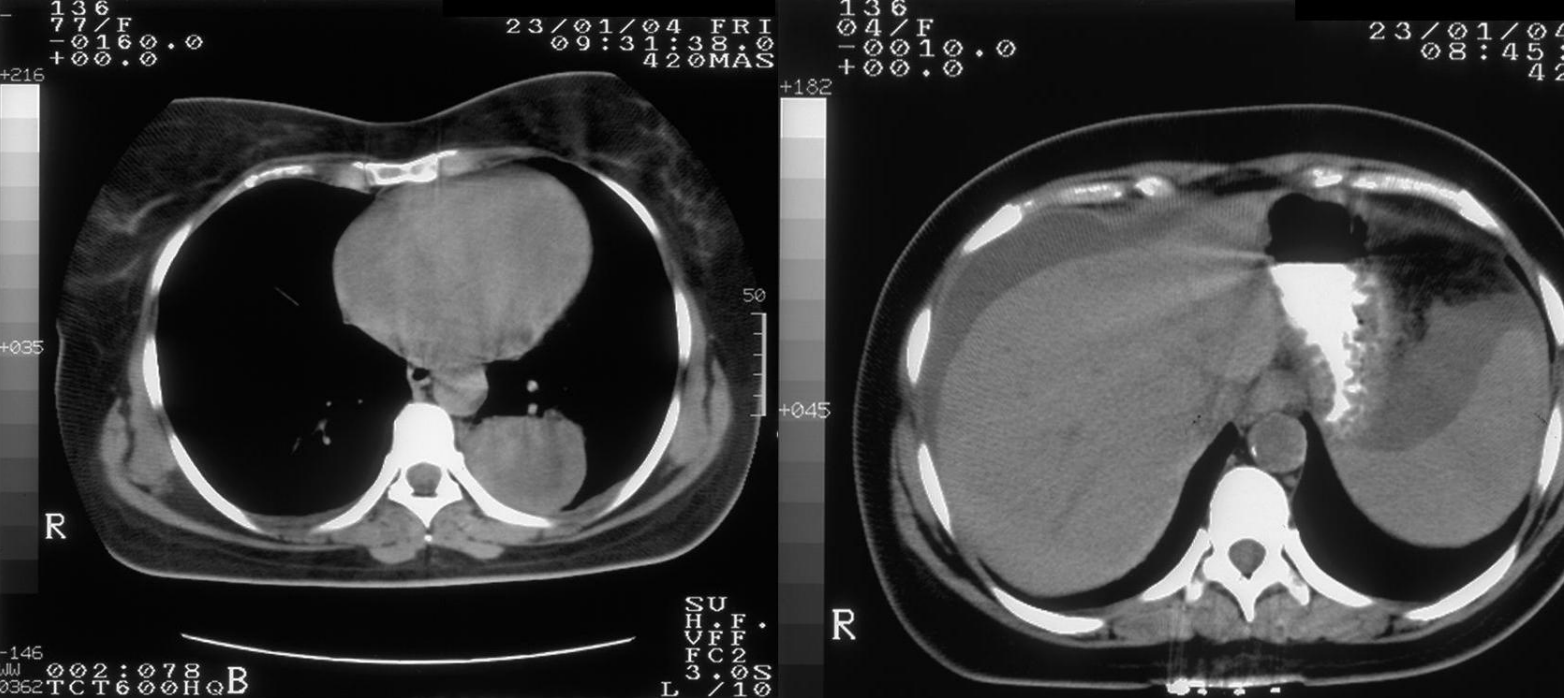
Thoracic and abdominal CT scans of the patient at initial presentation.

**Figure 2:**
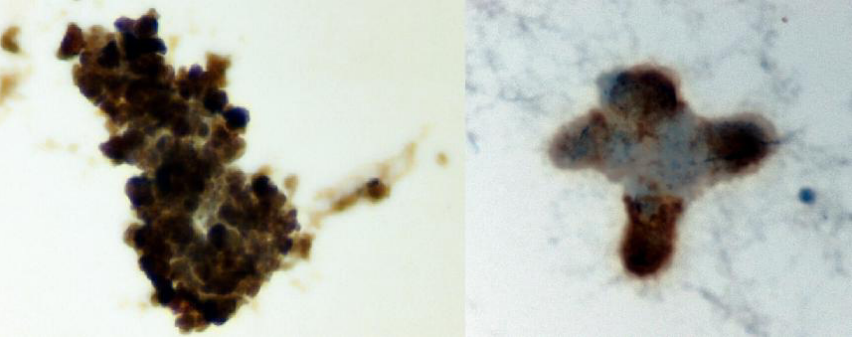
Melanoma cells showing strong positivity for Vimentin (left) and HMB-45 (right) (liquid based cytology – ThinPrep × 400).

A 43-year old female patient was referred to our department with the clinical diagnosis of ovarian malignancy. Abdominal CT scan revealed a left adnexal mass, moderate perihepatic and perisplenic ascitic fluid collection, retroperitoneal and pelvic lymph node enlargement and omental cake peritoneal seedings; thorax CT identified a paravertebral pleural cystic lesion with thick wall and serous liquid content (Fig. 1). Laboratory investigations showed a mildly elevated serum lactate dehydrogenase at 546 IU/L and CA 125 at 1420 IU/L. The patient's previous history was remarkable for a malignant melanoma of the left antecubital region removed surgically 9 years ago. Histopathology report at that time revealed a Breslow 2.36 mm, Clark's level IV, superficial spreading melanoma with a nodular phase growth pattern, with signs of regression and without ulceration. Elective left axillary lymph node dissection and intraoperative isolated limb perfusion of the left upper extremity with cisplatin, melphalan and dacarbazine was performed according to an investigational protocol at the time of initial presentation of the patient. Histopathologic examination showed no evidence of metastatic disease in the excised lymph nodes. After 2 years of well being the patient was lost from follow-up. In order to establish a definite diagnosis of the present clinical scenario, ultra-sound guided aspiration of the ascitic fluid and cytopathological examination was performed. Cytological morphology showed a cellular smear with a single cell population of large pleomorphic round undifferentiated pigmented malignant cells with moderate to abundant cytoplasm. The hyperchromatic nuclei showed great variation in size and contained large nucleoli. There was pigment both within the cytoplasm and in the background. Binucleate and multinucleated cells were also frequent. Immunocytochemistry studies revealed positivity for HMB-45, vimentin and S-100 (Fig. 2). The findings were identical to the initial specimen of the same patient and diagnostic of metastatic melanoma. Systemic cisplatin-based cytotoxic chemotherapy was initiated. After a short period of disease stabilization the patient developed brain metastases and died 8 months after the diagnosis of disease relapse.

## Discussion

Primary epithelial ovarian cancer (EOC) is the leading cause of death from gynecologic cancer [4]. The ovary is also a frequent site of secondary spread from extra-ovarian malignant neoplasms. Ovarian involvement most commonly occurs via contiguous spread from neighboring organs or via the peritoneal route. Most common primary tumors include gynecologic and gastrointestinal cancers [1,5]. Other malignancies, such as breast cancer and malignant melanoma involve the ovaries secondarily through the hematogenous route [2,3]. Ovarian metastasis is generally associated with a poor prognosis [1,2].

Melanoma involving the ovary is uncommon and it rarely presents clinically as an ovarian mass [6]. Ovarian involvement occurs in up to 20% of patients with melanoma in autopsy series, however premortem diagnosis is uncommon, mostly due to the fact that it is commonly associated with disseminated disease and is therefore clinically irrelevant [7]. Our patient had an intermediate risk, stage IIA (T3a) melanoma, thus a 36% risk of death at 10 years [8]. Adverse prognostic features included the presence of histologic regression, whereas age, sex and anatomic location of her primary lesion were favourable. However delayed disease recurrences have been observed as late as 27 years after initial diagnosis even in early stage melanomas [9]. Although 95% of disease recurrences in Stage III melanoma occur within 5 years, node-negative melanomas, thin or non-ulcerated lesions, younger age, as well as adjuvant treatment tend to correlate with delayed recurrences [10]. The time interval between the diagnosis of the primary melanoma and ovarian metastasis has ranged from months up to 18 years [3].

Most metastatic tumors involve both ovaries. On the contrary, ovarian metastases from melanoma are mostly unilateral [3], as in our patient. Women of reproductive age are more prone to metastatic ovarian involvement, which may be attributed to the higher blood flow to the premenopausal ovary [2]. The extremities are the most frequent primary localization of melanoma, secondarily involving the ovaries [3], as in our patient.

Cases of ovarian metastasis from melanoma published so far have been almost invariably diagnosed following surgical treatment [3,6,7,11]. Survival was poor despite aggressive surgical debulking with or without adjuvant therapy. A more favorable subset of patients with metastatic ovarian involvement included gynecologic [5] or colonic primaries [12], isolated ovarian metastasis [2], absence of extrapelvic or extra-abdominal disease [12] and complete surgical resection of metastatic disease [2,12]. In contrast to primary EOC, there is no proven value for cytoreductive surgery in women with cancer metastatic to the ovaries [2]. Surgery is generally indicated in terms of diagnostic laparotomy or palliative procedures in painful or obstructing metastatic lesions.

In our patient, the remote history of melanoma was ignored, considered irrelevant to the present clinical presentation. A diagnosis of advanced ovarian malignancy was suspected and a neoadjuvant taxane-platinum chemotherapy was proposed. In order to obtain pathologic diagnosis to guide further treatment, ascitic fluid cytology was performed. Neoplastic cells were identified with features consistent with the diagnosis of metastatic melanoma. The findings were identical to the initial specimen of the same patient and diagnostic of metastatic melanoma. Immunocytochemistry was positive for HMB-45, vimentin and S-100. S-100 and HMB-45 are the two most sensitive markers, being positive in 95% and 85% of melanoma cases respectively [3].

The fact that the cytopathologist was made aware of the previous history of melanoma was crucial; otherwise the clinical picture simulating ovarian cancer may have lead to a different therapeutic strategy. Even more challenging are the cases without an obvious history of melanoma. A regressed primary lesion may underlie such cases or the rare primary ovarian melanoma arising within a teratoma [3]. Another clue to an extra-ovarian origin in such clinical cases is the presence of metastases to sites not usually seen with primary ovarian cancer, such as the brain or skin [7]. In only one case so far, described by Moselhi et al [11], diagnosis was established preoperatively via ascitic fluid cytology. However, it was a case with evident pulmonary nodules and lytic bone lesions, which were highly unlikely to be due to EOC.

The elevated serum LDH was a hint in our patient and after establishing diagnosis it was helpful in assessing disease burden. Serum lactate dehydrogenase seems to be a simple yet quite powerful predictor of survival in patients with metastatic melanoma [13]. Tumor marker elevation was misleading and imaging studies were only indicative of a malignant process, but not conclusive. Serum S-100 might have been helpful if available, although it is of limited value in the metastatic setting [14].

Initial staging should evaluate thoroughly disease extent, in order to establish the diagnosis of potentially respectable metastatic disease. Surgical treatment for abdominal metastases of melanoma in one report significantly prolonged survival; however complete resection was only possible in one-third of the patients [15]. Unilateral salpingo-oophorectomy has been proposed as an appropriate treatment for metastatic melanoma involving the ovary, if there is no evidence of contralateral ovarian involvement or extraovarian spread [2,7]. In such cases of apparently resectable metastatic disease, preoperative screening for metastatic disease in other sites is crucial, either with conventional imaging or with PET scanning [16]. No postoperative adjuvant therapy is of proven benefit for improving survival [7,17]. In our patient the evidence of diffuse abdominal metastatic involvement rendered the disease irresectable and the therapeutic target was palliation.

## Conclusion

The present case illustrates the unpredictable and diverse natural history of malignant melanoma. It also highlights the importance of a previous history of melanoma in a patient presenting with signs of a second primary malignancy even after a long remission period. Certain parameters should be considered to establish a definite diagnosis and avoid unnecessary surgical intervention. Disease recurrence should always be taken in account, even after long periods of remission. Secondary ovarian involvement is associated with a poor prognosis and efforts should be made for adequate palliation. Pathologic diagnosis with non-invasive procedures is crucial in order to avoid unnecessary surgery. Surgical interventions may be undertaken only in selected cases of limited metastatic disease, where complete resection is expected.

## Consent

Written informed consent was obtained from the patient's husband for publication of this case report and any accompanying images. A copy of the written consent is available for review by the Editor-in-Chief of this journal.

## Competing interests

The authors declare that they have no competing interests.

## Authors' contributions

AB-conception and design, collection and assembly of data, analysis and interpretation of data, manuscript writing. RV-conception and design collection and assembly of data, analysis and interpretation of data. IK-collection and assembly of data, manuscript writing. DV-collection and assembly of data, editing. IA-collection and assembly of data, manuscript writing. CA-conception and design, analysis and interpretation of data, manuscript writing. All authors read and approved the manuscript.
